# Genes associated with inflammation and bone remodeling are highly expressed in the bone of patients with the early-stage cam-type femoroacetabular impingement

**DOI:** 10.1186/s13018-021-02499-y

**Published:** 2021-05-29

**Authors:** Guanying Gao, Ruiqi Wu, Rongge Liu, Jianquan Wang, Yingfang Ao, Yan Xu

**Affiliations:** Institute of Sports Medicine, Beijing Key Laboratory of Sports Injuries, Peking University Third Hospital, 49 North Garden Road, Haidian District, Beijing, 100191 China

**Keywords:** Femoroacetabular impingement, Gene, Inflammation, Bone remodeling

## Abstract

**Background:**

Recent studies have shown high expression levels of certain inflammatory, anabolic, and catabolic genes in the articular cartilage from the impingement zone of the hips with femoroacetabular impingement (FAI), representing an increased metabolic state. Nevertheless, little is known about the molecular properties of bone tissue from the impingement zone of hips with FAI.

**Methods:**

Bone tissue samples from patients with early-stage cam-type FAI were collected during hip arthroscopy for treatment of cam-type FAI. Control bone tissue samples were collected from six patients who underwent total hip replacement because of a femoral neck fracture. Quantitative real-time polymerase chain reaction (PCR) was performed to determine the gene expression associated with inflammation and bone remodeling. The differences in the gene expression in bone tissues from the patients with early-stage cam-type FAI were also evaluated based on clinical parameters.

**Results:**

In all, 12 patients with early-stage cam-type FAI and six patients in the control group were included in this study. Compared to the control samples, the bone tissue samples from patients with FAI showed higher expression levels of interleukin-6 (IL-6), alkaline phosphatase (ALP), receptor activator of nuclear factor-kB ligand (RANKL), and osteoprotegerin (OPG) (*P* < 0.05). IL-1 expression was detected only in the control group. On the other hand, there was no significant difference in IL-8 expression between the patients with FAI and the control group. The patients with FAI having a body mass index (BMI) of >24 kg/m^2^ showed higher ALP expression (*P* < 0.05). Further, the expression of IL-6 and ALP was higher in the patients with FAI in whom the lateral center-edge angle was >30° (*P* < 0.05).

**Conclusions:**

Our results indicated the metabolic condition of bone tissues in patients with early-stage cam-type FAI differed from that of normal bone in the femoral head-neck junction. The expression levels of the genes associated with inflammation and bone remodeling were higher in the bone tissue of patients with early-stage cam-type FAI than in the patients with normal bone tissue.

## Background

Femoroacetabular impingement (FAI), which can be divided into cam impingement and pincer impingement, is a major and common cause of hip pain [1]. It causes chondral injury and labral damage and may be involved in the etiology in up to 50% of hip osteoarthritis (OA) case s[2–5]. However, the impact of FAI on articular cartilage, subchondral bone, and joint biology at the cellular and molecular level is poorly understoo d[6]. Various studies have been conducted on the pathogenesis and pathologic process of hip OA caused by FA I[6–14]. Recent studies have shown high expression levels of certain inflammatory, anabolic, and catabolic genes in the articular cartilage from the impingement zone of the hips with FAI, representing an increased metabolic stat e[6, 13]. The metabolic changes were heightened by mechanical impingemen t[13]. The hip cartilage in patients with FAI has been reported to exhibit an OA phenotype in those with early-stage FAI, similar to that observed in patients with hip OA secondary to FA I[12]. All recent studies on the pathogenesis and pathologic process of hip OA caused by FAI focused on the cartilage or the synovial membrane. OA is characterized by complete organ failure that affects different tissues including cartilage, bone, and the synovial membrane. Changes in bone tissue in OA have long been considered secondary to cartilage degeneration; however, it is now suggested that the modification of the subchondral bone is one of the most significant causal pathophysiological events occurring in the cartilag e[15]. In addition, studies have shown that the mineral density of the subchondral bone in cam deformities is higher than that of normal subchondral bone in the peripheral region of the femoral hea d[16]. This phenomenon can also be observed in the subchondral bone in the anterosuperior acetabular are a[17, 18]. The increased subchondral stiffness may increase contact stresses in the joint tissues, leading to accelerated degeneration. At present, little is known about the molecular properties of bone tissue in the impingement zone of the hips with FAI.

We hypothesized that bone tissue from the impingement zone in patients with early-stage cam-type FAI exhibits tissue degeneration and has high expression levels of molecular markers of inflammation. Studying the properties of bone tissue from the cam-type impingement zone can help us better understand the mechanisms of early-stage cam-type FAI and the pathogenesis and pathologic process of hip OA caused by FAI. Hence, the purpose of this study was to evaluate the levels and properties of inflammatory markers in bone tissue from the head-neck junction in patients with early-stage cam FAI.

## Methods

### Patients and bone tissue samples

This study was approved by the institutional review board, and informed consent was obtained from all patients. Patients with OA, previous hip trauma, infection or osteonecrosis of the femoral head, or prior hip surgery were excluded from this study. Patients receiving any anti-inflammatory medications were also excluded. Twelve symptomatic patients with cam-type FAI were included in this study. All patients underwent a thorough and systematic physical examination, including specific tests previously described for diagnosing hip patholog y[19]. Flexion, adduction, and internal rotation (FADIR) or flexion abduction external rotation (FABER) tests were considered positive if hip or groin pain was elicited when the hip was placed in 90° of flexion and then adduction and internal rotation or flexion, abduction, and external rotation applie d[20]. Supine anteroposterior (AP) pelvis radiographs, Dunn view (45 degrees) radiographs, and CT and MRI scans were preoperatively obtained for all patients with FAI. The alpha angle and lateral center-edge angle (LCEA) were calculated as described previousl y[21, 22].

Bone tissue samples from patients with early-stage cam-type FAI were collected during hip arthroscopy for the treatment of cam-type FAI. Control bone tissue samples were collected from six patients who underwent total hip replacement because of femoral neck fracture. Control bone tissue samples were collected from the anterolateral femoral head-neck junction of the hips, corresponding to the same area from which samples were obtained in the case of the hips with cam-type FAI. Gene expression in the samples from 12 hips with a diagnosis of cam-type FAI was compared with that in the six control samples of bone from the hips without OA or FAI. The differences in the gene expression in bone tissues from the patients with early-stage cam-type FAI were also evaluated based on clinical parameters.

### Isolation of RNA and quantitative real-time polymerase chain reaction

After the samples were taken out of the operating room, the remaining connective tissue was removed immediately under RNase-free conditions. Then, the bone tissue was frozen using liquid nitrogen and then ground into powder for 5 min with a liquid nitrogen grinder (Cryomill, Verder Shanghai Instruments and Equipment, Shanghai, China). The powder was then soaked in TRIzol Reagent (Invitrogen, Carlsbad, CA, USA), and each sample was stirred for 30 s with a homogenizer to fully lyse the sample. RNA was extracted according to the manufacturer’s protocols. Briefly, chloroform was added to the samples, and centrifugation was performed. Then, the supernatant was transferred to a tube containing isopropanol then rinsed with 75% ethanol, and the RNA was collected by centrifugation.

RevertAid RT Reverse Transcription Kit (Thermo Fisher Scientific, Boston, MA, USA) was used for reverse transcription of RNA, and cDNA was obtained according to the instructions. In brief, RNase-free water was mixed with the sample RNA and random primers to make up a total volume of 12 μl, and the reaction was performed at 65 °C for 5 min. Then, add 8 μl of mixed reaction buffer, dNTP mix, Revert Aid RT, and RNase inhibitor, reacted at 42 °C for 60 min, and react at 70 °C for 5 min. The samples were stored at −20°.

Quantitative real-time polymerase chain reaction (qPCR) was performed with 15 μl of reaction mixture containing 6.5 μl of the sample, 7.5 μl of SHBR Green PCR Master Mix (Invitrogen, Carlsbad, CA, USA), and 1 μl of primers (Sangon Biotech, Shanghai, China). The primer sequences of interleukin-1beta (IL-1β), IL-6, IL-8, alkaline phosphatase (ALP), receptor activator of nuclear factor-kB ligand (RANKL), osteoprotegerin (OPG), and glyceraldehyde 3-phosphate dehydrogenase (GAPDH) are given below. GAPDH was used as the reference gene control. Real-time RT-PCR was performed with the StepOnePlus Real-Time PCR System (Applied Biosystems, Foster City, CA, USA). The relative change in the gene expression was determined using the 2−ΔΔCt method.

### qPCR primers

The primer sequences used were as follows: GAPDH—forward GCACCGTCAAGGCTGAGAAC, reverse TGGTGAAGACGCCAGTGGA; IL-1β—forward GCCAGTGAAATGATGGCTTATT, reverse AGGAGCACTTCATCTGTTTAGG; IL-6—forward CACTGGTCTTTTGGAGTTTGAG, reverse GGACTTTTGTACTCATCTGCAC; IL-8—forward GAAGGTGCAGTTTTGCCAAG, reverse TGTGGTCCACTCTCAATCCTC; RANKL—forward TTACCTGTATGCCAACATTTGC, reverse TTTGATGCTGGTTTTAGTGACG; ALP—forward CTGGTACTCAGACAACGAGATG, reverse GTCAATGTCCCTGATGTTATGC; OPG—forward GAAACGTTTCCTCCAAAGTACC, reverse CTGTCTGTGTAGTAGTGGTCAG.

### Statistics

The nonparametric Mann-Whitney U test was used for comparisons between the cam-type FAI group and the control group. The different gene expression profiles in the cam-type FAI or control group were non-normal distributed. All experiments were performed at least thrice. SPSS version 20.0 (SPSS Inc., Chicago, IL, USA) was used to process the data. Significance was set at *P* < 0.05.

## Results

As shown in Table 1, 12 symptomatic cam-type FAI patients (mean age, 33.8 years; age range, 16–65 years; 4 males and eight females) and six control participants (mean age, 55.3 years; age range, 52–59 years; 2 males and four females) were included in this study. The mean duration time of symptoms in FAI patients was 13.9 months (range, 1–48 months). The body mass index (BMI) in the cam-type FAI group and the control group were 23.0 (range, 17.6–27.3) and 24.5 (range, 20.3–27.3), respectively. The results of the FADIR test as evaluated by the treating physician were positive in all patients. The physician obtained a positive FABER test result in 10 (83.3%) patients. The mean alpha angle of all cam-type FAI patients was 67.5° ± 6.5°, and the mean LCEA was 29.3° ± 3.8°. Demographic data for symptomatic cam-type FAI patients and the control group are also shown in Table 1.

**Table 1:** Demographic characteristics of patients

Parameter	FAI group	Control group
Number	12	6
Age, years, mean (range)	33.8 (16–65)	55.3 (52–59)
Gender		
Male	4 (33.3%)	2 (33.3%)
Female	8 (66.7%)	4 (66.7%)
BMI, kg/m^2^, mean (range)	23.0 (17.6–27.3)	24.5 (20.3–27.3)
Positive FADIR	12 (100%)	
Positive FABER	10 (83.3%)	
Duration of symptoms (range)	13.9 (1–48)	
Alpha angle, degree (range)	67.5 (58.9–75.8)	
LCEA, degree (range)	29.3 (25.3–36.5)	
Diagnosis		
Cam-type FAI	12 (100%)	
Pincer-type FAI	6 (50%)	
Labral tear	11 (91.7%)	
Femoral neck fracture		6 (100%)

*Note*: unless otherwise specified, data are numbers of patients, with percentages in parentheses

**Fig. 1:**
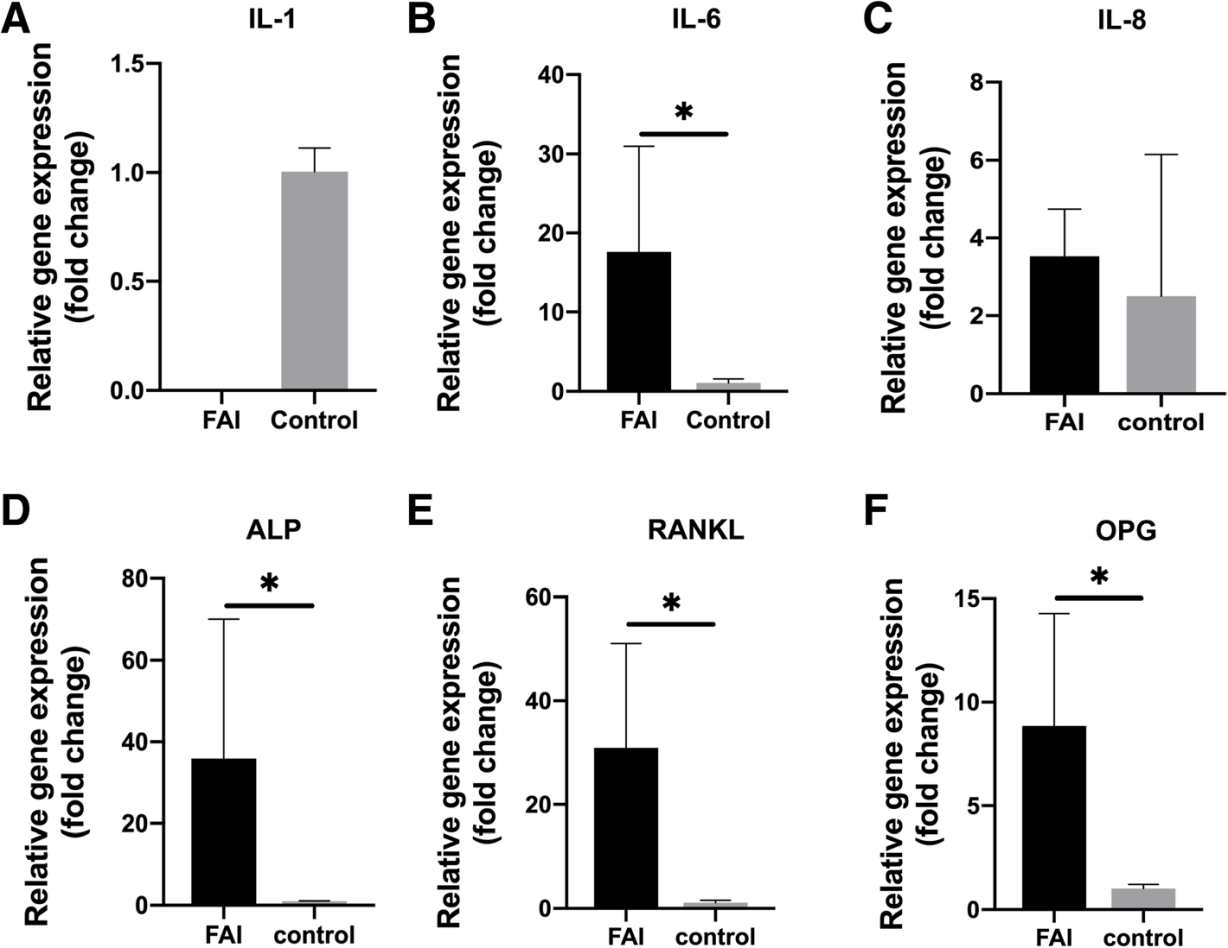
Differences in the gene expression in bone tissues from patients with early-stage cam-type FAI and controls

The inflammatory factors, osteoblasts, and osteoclast-related genes regulating normal bone modeling and remodeling in all patient samples were evaluated. qPCR results showed that the expression levels of all inflammatory cytokines, except IL-1 and IL-8, and osteogenic and osteoclast-related genes in the bone tissue samples of FAI patients were increased in comparison with the corresponding levels in the control group (Fig. 1).

The mRNA expression level of IL-6 was significantly higher in the FAI patients (*P* < 0.05) (Fig. 1B). In contrast, the inflammatory factor IL-1 was only expressed in the control group, and was undetectable in FAI samples. The gene expression for ALP was almost 40-fold higher than that in the control group (*P* < 0.05) (Fig. 1D), while the expressions of RANKL and OPG were also significantly upregulated, with the expression of RANKL being approximately 30-fold higher than that in the control group (*P* < 0.05) (Fig. 1E). OPG expression was also significantly higher than that in the control group (*P* < 0.05) (Fig. 1F).

**Fig. 2:**
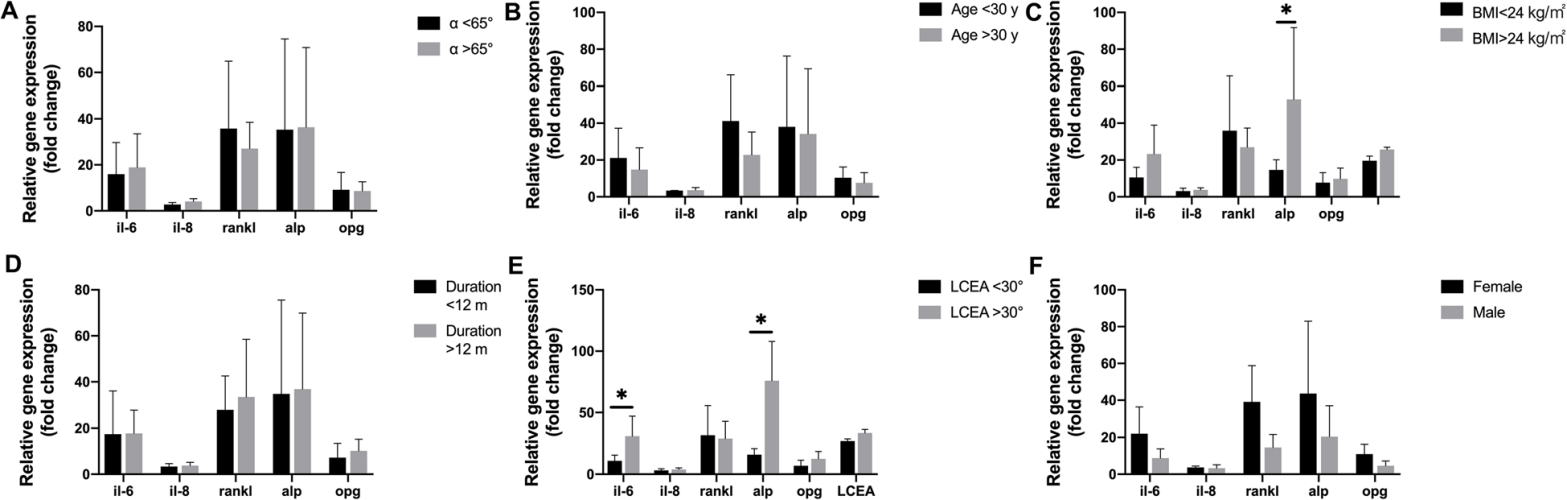
Gene expression in bone tissues from patients with early-stage cam-type FAI based on clinical parameters

The gene expression was compared according to the clinical parameters of patients, as shown in Fig. 2. The gene expression in bone tissue from the cam zone was compared between patients with an alpha angle of >65° and those with an alpha angle of <65°. There was no significant difference in the gene expression between these two groups (Fig. 2A). Similarly, there was no statistical difference in the gene expression between patients older than 30 years and those younger than 30 years (Fig. 2B). Comparison of FAI patients with BMI greater than 24 kg/m^2^ and less than 24 kg/m^2^ showed a significant difference in the expression of ALP (*P* < 0.05) (Fig. 2C), and FAI patients with BMI > 24 kg/m^2^ showed higher ALP expression. There was no significant difference in the gene expression between patients whose duration of symptoms was more than 1 year or less than 1 year (Fig. 2D). Comparison of patients whose LCEA was greater than 30° and less than 30° showed that the expression levels of IL-6 and ALP were higher in patients with LCEA > 30° (*P* < 0.05) (Fig. 2D). The average expression of IL-6, RANKL, ALP, and OPG genes in women was also higher than that in men, but the differences were not significant (Fig. 2F).

## Discussion

In this study, we found that bone tissue from the impingement zone of cam-type FAI expressed high levels of certain inflammatory, anabolic, and catabolic genes and genes regulating normal bone modeling and remodeling in comparison with normal bone tissue. Bone tissue from the impingement zone of cam-type FAI expressed higher levels of ALP, IL-6, OPG, and RANKL. IL-1 was only expressed in the control group, while it was undetectable in FAI samples. There was no significant difference in the expression of IL-8 between the FAI and control groups. FAI patients with a BMI of >24 kg/m^2^ showed higher ALP expression (*P* < 0.05). The expression levels of IL-6 and ALP were higher in FAI patients with an LCEA of >30° (*P* < 0.05).

The articular cartilage from the impingement zone of the hips with FAI is known to express higher levels of certain markers of inflammation and degeneratio n[6, 9–12]. Hashimoto et al .[6] proved that the mRNA expression of chemokine (IL-8, CXCL1, CXCL3, CXCL6, CCL3, and CCL3L1), matrix-degrading (matrix metalloproteinase [MMP]-13 and ADAMTS-4), and structural matrix (collagen, type II, alpha [COL2A1], and aggregan [ACAN]) genes was higher overall in the cartilage from the hips with FAI than in the hips with OA and normal controls. Haneda et al .[11, 12] proved that cartilage in the cam FAI group showed higher expression of the inflammatory molecules IL-1β, MMP-13, ADAMTS-4, and COL2. Speirs et al .[9] conducted an indentation test and histological analysis and proved that the cartilage from the cam deformity showed severe degeneration in terms of mechanical behavior and composition changes, and was consistent with OA. Wagner et al .[10] analyzed the cartilage in the aberrant nonspherical portion of the femoral head in young patients with an impingement conflict and proved that the hyaline cartilage in these areas showed clear degenerative signs similar to the findings in the osteoarthritic cartilage. However, all of these studies focused on the cartilage or synovial membrane of FAI patients, and little is known about the molecular properties of subchondral bone tissue from the impingement zone of the hips with FAI. Changes in the subchondral bone in the impingement zone could also be a component of the early events leading to the clinical stage of the degeneration. Subchondral bone alterations may actually precede cartilage changes as assessed in different animal OA models,[15] which may also occur in FAI. Bone formation and bone resorption both occur in the bone remodeling process. The molecular triad of RANKL, receptor activator of nuclear factor-kB (RANK), and OPG play essential roles not only in bone formation but also in bone resorption. RANKL/RANK signaling regulates osteoclast formation, activation, and survival in normal bone modeling and remodeling and in a variety of pathologic conditions characterized by increased bone turnove r[23]. OPG protects bone from excessive resorption by binding to RANKL and preventing it from binding to RAN K[23]. Thus, the relative concentration of RANKL and OPG in the bone is a major determinant of bone mass and strengt h[23–25]. The chemokines IL-1β, IL-6, and IL-8, which are produced by bone marrow stromal cells from subchondral bone, participate in the mechanisms directly or indirectly causing cartilage destruction and bone remodelin g[26–28]. ALP is also regarded as an indicator of bone formatio n[29]. Therefore, we compared ALP, IL-1β, IL-6, IL-8, OPG, and RANKL between bone tissue from the head-neck junction in patients with cam FAI and normal bone in the corresponding area to identify the molecular properties of bone in the cam zone.

The etiology of primary FAI remains controversial. Both genetic and acquired causes have been postulated and studie d[30]. Recent studies have suggested that genetic factors may play a role in the development of FAI, and several studies have supported the concept that the formation of cam lesions occurred with repetitive injury to the proximal femoral physis during a critical period of developmen t[30–32]. In this study, bone tissue from the impingement zone of cam-type FAI expressed higher levels of ALP, IL-6, IL-8, OPG, and RANKL. The higher levels of IL-6 and IL-8 may be related to the inflammatory reaction caused by mechanical impingement. High levels of OPG and RANKL expression suggested the presence of bone remodeling. We thought the mechanical impingement in the cam zone activated bone formation and bone resorption. Repeated impingement and bone remodeling may cause bone hyperplasia in the cam zone. However, it was difficult to distinguish whether cam formation was induced by the impingement, or the inflammatory reaction and bone remodeling induced by impingement caused congenital development of cam. It was difficult to identify the cause and the result. Additional research is needed to identify the etiology of FAI. In addition, IL-1 was only expressed in the control group, while it was undetectable in FAI samples. This phenomenon was similar to the findings for cartilage from the femoral head-neck junction in FAI patients in comparison with the normal cartilag e[6].

In addition, we evaluated the differences in the gene expression in bone tissues from the patients with early-stage cam-type FAI categorized on the basis of clinical parameters. Chinzei et al .[13] evaluated the gene expression in FAI synovial tissues from patients who had an alpha angle of >60° and compared the findings with those in patients who had an alpha angle of <59°. The two alpha angle groups showed no significant differences in the gene expression in synovial tissue. However, the mRNA expression of *COL1A1* was significantly higher in labral tissue samples from the group with the smaller alpha angles (*p* < 0.05). The mRNA expression of ACAN and ADAMTS-4 was significantly higher in the cartilage samples from the group with the larger alpha angles (*p* < 0.01). In our study, the gene expression of bone tissue from the cam zone was compared between patients with an alpha angle of >65° and patients who had an alpha angle of <65°. There was no significant difference in the gene expression in the two groups. However, FAI patients with BMI > 24 kg/m^2^ showed higher ALP expression (*P* < 0.05), and the expression levels of IL-6 and ALP were higher in FAI patients who had LCEA > 30° (*P* < 0.05) in this study. Patients with higher BMI and LCEA may have more severe mechanical impingement, which may lead to higher expression levels of IL-6 and ALP. Further research is needed to explore the cause of this phenomenon.

This study had several limitations. Firstly, although one important advantage of this study was the availability of control hip bone tissue samples from patients without OA, which provided baseline data for comparison with bone tissue from the hips with cam-type FAI and normal bone tissue, the sample size of the control group was relatively small. Secondly, the age distributions of the FAI and control groups did not match. Most young patients with femoral neck fractures preferred to undergo internal fixation while older patients with FAI always underwent combined OA, which could have influenced the outcomes. Third, we obtained bone samples from the anterolateral femoral head-neck junction in the area of mechanical impingement. The molecular characteristics in other normal zones of the bone of the hip were unknown.

## Conclusion

In conclusion, our results indicated that the metabolic condition of bone tissues in patients with early-stage cam-type FAI was different from that of normal bone in the femoral head-neck junction. The expression of genes associated with inflammation and bone remodeling was higher in the bone tissue from the early-stage cam-type FAI in comparison with normal bone tissue.

## Data Availability

Not applicable.

## Abbreviation

FAI: Femoroacetabular impingement
